# Postmortem toxicology findings from the Camden Opioid Research Initiative

**DOI:** 10.1371/journal.pone.0292674

**Published:** 2023-11-01

**Authors:** Dara M. Kusic, Jessica Heil, Stefan Zajic, Andrew Brangan, Oluseun Dairo, Stacey Heil, Gerald Feigin, Sherri Kacinko, Russell J. Buono, Thomas N. Ferraro, Rachel Rafeq, Rachel Haroz, Kaitlan Baston, Elliot Bodofsky, Michael Sabia, Matthew Salzman, Alissa Resch, Jozef Madzo, Laura B. Scheinfeldt, Jean-Pierre J. Issa, Jaroslav Jelinek

**Affiliations:** 1 Research, Coriell Institute for Medical Research, Camden, New Jersey, United States of America; 2 Clinical Research Office, Cooper University Health Care, Camden, New Jersey, United States of America; 3 Coriell Institute for Medical Research, Camden, New Jersey, United States of America; 4 Office of the Medical Examiner, Gloucester County Health Department, Sewell, New Jersey, United States of America; 5 Forensic Toxicology, NMS Labs, Horsham, Pennsylvania, United States of America; 6 Biomedical Sciences, Cooper Medical School of Rowan University, Camden, New Jersey, United States of America; 7 Department of Emergency Medicine, Cooper University Health Care, Camden, New Jersey, United States of America; 8 Cooper Medical School of Rowan University, Camden, New Jersey, United States of America; 9 Neurological Institute, Cooper University Health Care, Camden, New Jersey, United States of America; 10 Anesthesiology, Cooper University Health Care, Camden, New Jersey, United States of America; University of New Mexico Health Sciences Center, UNITED STATES

## Abstract

The United States continues to be impacted by decades of an opioid misuse epidemic, worsened by the COVID-19 pandemic and by the growing prevalence of highly potent synthetic opioids (HPSO) such as fentanyl. In instances of a toxicity event, first-response administration of reversal medications such as naloxone can be insufficient to fully counteract the effects of HPSO, particularly when there is co-occurring substance use. In an effort to characterize and study this multi-faceted problem, the Camden Opioid Research Initiative (CORI) has been formed. The CORI study has collected and analyzed post-mortem toxicology data from 42 cases of decedents who expired from opioid-related toxicity in the South New Jersey region to characterize substance use profiles. Co-occurring substance use, whether by intent or through possible contamination of the illicit opioid supply, is pervasive among deaths due to opioid toxicity, and evidence of medication-assisted treatment is scarce. Nearly all (98%) of the toxicology cases show the presence of the HPSO, fentanyl, and very few (7%) results detected evidence of medication-assisted treatment for opioid use disorder, such as buprenorphine or methadone, at the time of death. The opioid toxicity reversal drug, naloxone, was detected in 19% of cases, but 100% of cases expressed one or more stimulants, and sedatives including xylazine were detected in 48% of cases. These results showing complex substance use profiles indicate that efforts at mitigating the opioid misuse epidemic must address the complications presented by co-occurring stimulant and other substance use, and reduce barriers to and stigmas of seeking effective medication-assisted treatments.

## Introduction

Since the 1990s, the United States (US) has seen three waves of the opioid use epidemic, beginning with increases in toxicity cases involving prescription opioids, followed by heroin and, more recently, fentanyl and fentanyl analogs otherwise known as highly potent synthetic opioids (HPSO) [1–5]. Although the rate of opioid toxicity death due to heroin decreased in recent years, the rate due to HPSO, including fentanyl, is rising sharply, increasing by 56% in just one year from 2019 to 2020 [6]. Fentanyl and fentanyl analogs have become even more prevalent since the start of the COVID-19 pandemic [7–11]. In 2021, fentanyl overtook heroin for the first time as the most common opioid agent in toxicology reports [12].

In addition to the increase in the number of toxicity events involving HPSO, there has also been a nationwide increase in the number of toxicity deaths that involve both HPSO and stimulants [7, 13–15]. Combining cocaine and other stimulants with fentanyl is on the rise nationally, including South New Jersey, and may contribute to the rise in HPSO-related toxicity deaths [16, 17]. Many individuals who use opioids chronically and those with opioid use disorder (OUD) have experienced a toxicity event or have been exposed to toxicity response training using the anti-opioid-toxicity drug, naloxone. Some evidence suggests that individuals who use stimulants and are not intending or not expecting to take opioids or synthetic opioids are less likely to be knowledgeable of and prepared for an opioid-related drug toxicity event [18–20].

Whereas an increasing prevalence of HPSO and a lack of toxicity preparedness are risk factors for opioid-related toxicity death, a more comprehensive understanding of drug toxicity risk is still needed. The Camden Opioid Research Initiative (CORI) is a multi-armed research study including a biobank resource of biospecimens collected from individuals who died from opioid-related drug toxicity (CORI Biobank) [21]. As part of this initiative, we analyzed toxicology reports of postmortem blood samples collected from medically-confirmed opioid toxicity decedents in the South New Jersey region.

## Methods

Samples were collected from decedents in Camden or Gloucester Counties, New Jersey, US, between March 2019 and April 2021. Deaths evaluated and affirmed by the Gloucester County medical examiner’s office to have been due to opioid-related drug toxicity were considered for inclusion in the study. Inclusion criteria specify that the next of kin must be identifiable and able to be contacted, over age 18 years, not incarcerated, English language proficient, and agree in writing to the sample’s submission to the study while the decedent is still in the custody of the medical examiner; any sample not meeting these conditions was excluded.

The study described in this protocol is not classified as Human Subjects Research because it involves the collection of blood samples after death; however, the CORI Biobank is overseen by the Coriell Institute of Medical Research Institutional Review Board (IRB) to ensure that the rights of the donor and their next of kin are appropriately respected. The study was conducted according to the guidelines of the Declaration of Helsinki, and the study protocol, #R163, was reviewed and approved by the Coriell IRB.

After assessing the decedent’s death was due to opioid toxicity, the medical examiner collected a sample of femoral blood in two vials containing sodium fluoride (NaF). The vials were then shipped to NMS Labs (Horsham, Pennsylvania, US) for expanded testing upon written consent from the decedent’s next of kin. Expanded toxicology testing at NMS involved two panels, 8092B (Forensic Postmortem Prescription Drugs Screen) and 8054B (NMS TotalTox^TM^ Panel), to identify fentanyl, fentanyl derivatives, synthetic cannabinoids, synthetic benzodiazepines, and other synthetic opioids that may contribute to opioid-related toxicity deaths. The full list of 77 compounds detected in our cases by the toxicology panels, including their classification for purposes of analysis and reporting in this study, and data on detected sample concentrations is shown in S1 Table. A full listing of the more than 360 analytes tested by the two panels is included in S1 File, and raw data of testing results used for analysis is included in S2 File. In total, 42 next of kin consented to donation to this study, and all samples were successfully assayed using the two expanded panels.

## Results

The study sample consisted of 35 male decedents (83%) and seven female decedents (17%). The median age of all decedents was 35.5 years with a range [min, max] of [21, 58] years. Race and ethnicity information about the decedents was not provided by the next of kin, and information other than gender and age were not provided by the Gloucester County medical examiner’s office for the purpose of characterizing the donor demographics. The average time elapsed from the date of death provided by the medical examiner to the date of blood collection was 0.26 days, in the range of 0–2 days.

The toxicology analysis revealed the presence of multiple compounds in postmortem blood samples from every decedent, with a median of nine compounds per sample (range 5–19). Eighteen of the compounds represent pairs of active drugs/analytes and respective metabolites, such as fentanyl-norfentanyl, where fentanyl is the un-metabolized HPSO, and norfentanyl is its metabolite. One compound, ethylecgonine, is formed when alcohol and cocaine are used simultaneously and was also reported in our screen (S1 Table). Table 1 shows the frequency of detection for each compound classification.

**Table 1:** Frequency of detected compound classes. Detected compound classes in 42 postmortem samples of femoral blood collected from individuals who died from opioid-related toxicity and submitted to the CORI Biobank. A full list of the 77 detected compounds with their individual detection frequency, detection limits, and classification can be found in S1 Table.

Compound Classification	Detected Frequency (N_max_ = 42)	Percent Frequency
Stimulant	42	100
Opioid HPSO	41	98
Sedative	20	48
Opioid non-HPSO	16	38
Non-opioid analgesic	15	36
Antidepressant/Antianxiety	13	31
Opioid toxicity reversal	8	19
Anticonvulsant	7	17
MOUD	3	7
Dissociative	2	5
Psychoactive bath salt	2	5
Antibiotic	1	2
Antimalarial	1	2
Bronchiodilator/Xanthine	1	2
Cardiovascular Medication	1	2
Cholinesterase inhibitor	1	2
PDE5 Inhibitor	1	2

The compound class detected with the greatest frequency, in all 42 decedent samples, was stimulants, which includes commonly consumed substances of caffeine and nicotine, and though these are not substances typically considered subject to substance use disorder, they may contribute to an additive effect when combined with other simulants. The most common stimulant was caffeine, detected in 38 samples (90%), followed by nicotine and its metabolite, cotinine, detected in 34 samples (81%). Cocaine and/or its metabolites were detected in 13 (31%) of the samples.

Next in frequency, the class of HPSO compounds including fentanyl and fentanyl metabolites were found in 41 (98%) of 42 postmortem blood samples. Median concentration of un-metabolized fentanyl was 16 ng/ml (detected range 1–73 ng/ml) and median concentration of norfentanyl was 2 ng/ml (detected range < 1–20 ng/ml). Other non-fentanyl HPSO were detected in seven (17%) of the samples, and free morphine was detected in 14 (33%) of the samples; of the 14 samples that tested positive for free morphine, nine registered sub-median fentanyl concentrations. The one case with no detectable fentanyl or fentanyl metabolites had a particularly high concentration of methadone. Opioids not classified as HPSO, which include codeine, morphine, and oxycodone, were detected in 16 (38%) of the samples as the fourth most frequent class of compounds, followed by non-opioid analgesics such as ibuprofen and acetaminophen in 15 (36%) of the samples. Antidepressant and antianxiety compounds were detected in 13 (31%) of the samples.

Medications to counteract opioid toxicity or to treat opioid addiction were among the least frequently detected compound classes. The opioid antagonist, naloxone, used to reverse the effects of opioid toxicity, was detected in eight (19%) of the samples. Available data on the decedents does not include the time at which naloxone had been administered relative to intake of the fatal dose of opioids or to the time of death. Most strikingly, just three (7%) of the samples showed evidence of medication used in the treatment of OUD (MOUD), which include methadone and buprenorphine. The MOUD, buprenorphine, was detected in one of these three samples, and methadone in the other two samples. One sample testing positive for methadone, also the lone sample without evidence of fentanyl, showed a high methadone concentration of 1400 ng/ml. Death due to methadone toxicity has been reported to occur at a concentration of 957 ng/ml, and the high concentration of methadone may have contributed to the fatality in this case, given the absence of HPSO [22].

**Fig 1:**
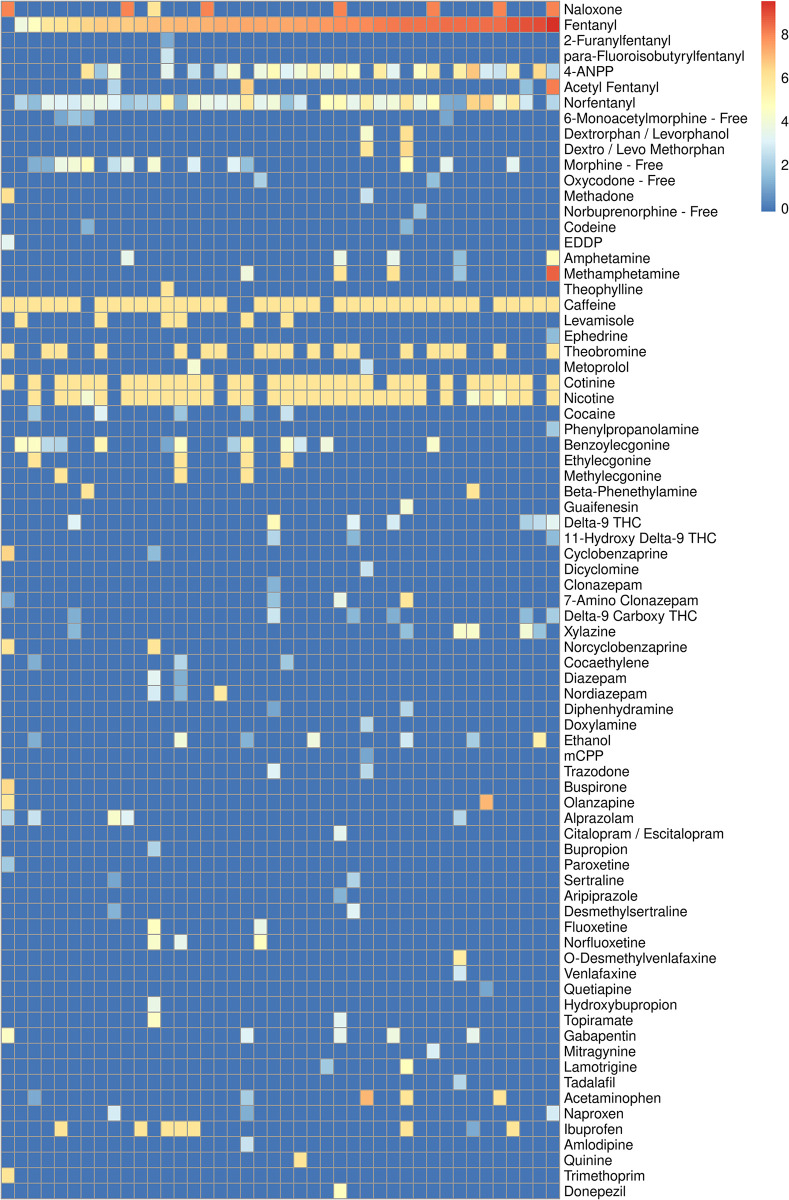
Heat map of detected compounds. Drug screen results in 42 postmortem samples of femoral blood collected from individuals who died from opioid-related toxicity and submitted to the CORI Biobank. Cases, ordered by ascending concentrations of fentanyl, are in columns. Toxicology screen compounds are in rows. The color scale shows the log_2_ fold-increase of the compound normalized to the detection limit. The dark blue cells represent undetected compounds.

Highlighted by the ubiquitous presence of stimulants in postmortem blood among the donors to the CORI biobank, the toxicology results document a high prevalence of co-occurring substance use profiles that accompany opioid use. Specifically, among commonly-recognized substances subject to abuse, 13 (31%) of the samples tested positive for cocaine, any sedative including benzodiazepine anxiolytics were found in 13 (31%), other benzodiazepines in seven (17%), amphetamine and/or methamphetamine in six (14%), alcohol or alcohol metabolites in eight (19%), and cannabinoid compounds in seven (17%) of the samples. Fig 1 plots the relative concentrations of each detected compound. S1 Fig plots the frequency of cases, in descending order, for each class of detected compound shown in Table 1 and annotated in S1 Table. The full list of compounds tested in the two expanded toxicology panels can be found in S1 File, and the double-blinded raw data set from the toxicology results can be found in S2 File.

Co-occurring substance use was prevalent in both sexes. Stratification of the toxicology data by sex or age did not reveal statistically significant differences between male and female or young and old decedents (p values of 0.7 and 0.3 for sex and age, respectively, according to Mann-Whitney-Wilcoxon Tests performed in R [23]). Within all stratifications, the most common class of compounds detected was stimulants, followed by HPSO, and then by sedatives.

## Discussion

Considering only stimulants, sedatives, dissociatives, and psychoactive bath salts as those compound classes associated with SUD, while excluding fentanyl and HPSO, non-HPSO opioids, as well as commonly-consumed caffeine, nicotine, and its metabolite, cotinine, from the stimulants, a large majority of 36 (86%) of the samples showed a profile of co-occurring substance use, while just three (7%) of the samples showed evidence of MOUD in a possible effort to treat opioid use disorder. The prevalence of co-occurring substance use and the low rate of MOUD observed in our cases are consistent with literature reports on individuals with OUD [24]. For example, a recent study conducted by the US Veterans Health Administration found that the majority of those with OUD also had at least one non-opioid substance use disorder (SUD). Further, individuals with OUD and at least one non-opioid SUD were less likely to enroll in medication-assisted OUD treatment [25, 26]. More broadly, a recent report on the opioid crisis indicates that co-occurring substance use is ubiquitous among all individuals who misuse opioids, and concludes that trends for other substances should be monitored during treatment [27].

Among the stimulants, cocaine and its analytes and metabolites were detected in 13 (31%) of the cases. Cocaine can lead to relapse and attrition from treatment programs for OUD [28]. Accounting for nicotine and its metabolite, cotinine, detected in 34 (81%) of the samples, among the stimulants considered for SUD, the incidence of co-occurring substance use rises to 41 (98%) of the cases. Nicotine, like many opioids, is metabolized by cytochrome P450 enzymes and interacts with opioids to contribute to an additive effect concomitant with deleterious health conditions when used concurrently [29–31]. Moreover, concurrent use of opioids and nicotine can make abstinence from either substance more difficult [32, 33].

The high incidence of caffeine, a stimulant also metabolized by cytochrome P450 enzymes, detected in 37 (88%) of the samples, may be due to commonly consumed caffeinated beverages, though there is literature to support that caffeine is introduced into the illicit opioid supply in an effort to make the drug less lethal in addition to simply adding bulk for increased profit [34–37]. Accounting for caffeine among the stimulants, all 42 (100%) of the cases showed evidence of co-occurring stimulant and opioid use.

Fentanyl was the dominant HPSO among this class of compounds in the CORI Biobank study. In the past decade, deaths from opioid-related toxicity have been driven by an increase in HPSO use [38, 39], a trend that is supported by the toxicology reports collected in this study, finding fentanyl and norfentanyl concentrations in 41 (98%) of the 42 postmortem blood samples, an incidence rate higher than the national average of 88% for opioid-related deaths involving HPSO reported by the National Institute on Drug Abuse for 2021 [7]. Overall deaths due to drug toxicity have risen sharply within recent years in the Northeastern United States, including New Jersey, and have increased exponentially together with arrests for possession of fentanyl, in contrast to other states where drug-related deaths have plateaued and criminal seizures of fentanyl have been fewer [40, 41]. While national and regional trends consistent with toxicological evidence presented here suggest that fentanyl is the likely compound implicated in all but one of the fatal toxicity cases studied by the CORI Biobank initiative, the amount of added toxicity when fentanyl combines with other compounds present is unclear [42–46]. The source of the fentanyl, whether combined with heroin or prepared in another format, is similarly unclear; however, we observe that 14 (34%) of the 41 samples with HPSO concentrations also had detectable morphine and 6-monoacetylmorphine, metabolites of heroin, suggesting that, in most cases, whereas a decedent may have sought heroin, fentanyl was the primary cause of death [47].

Presuming that fentanyl is a likely causative factor in most of these drug toxicity deaths, the lethal dosage is difficult to estimate given the uncertain timing of fentanyl exposure relative to the time of toxicity reaction and to the pharmacokinetics for chronic use or OUD. Short-acting opioids are cleared from the bloodstream in 2–4 days; however, the metabolism and clearance of fentanyl, particularly in individuals exposed to opioids chronically, require a much longer time [48]. For example, in individuals with OUD, norfentanyl, the major metabolite of fentanyl, requires 13 days on average to fall to undetectable levels in the bloodstream after the last exposure [48].

Among sedatives, the third most common class of compounds detected after stimulants and HPSO in the CORI Biobank, benzodiazepines were detected in 17% of cases, and though there are reports of this class of drugs appearing in the illicit opioid supply and in postmortem samples from drug-related deaths, it is unclear whether the benzodiazepine was combined with an opioid or taken separately [49, 50]. Xylazine, a powerful veterinary tranquilizer for which a lethal dose in humans is not established, was detected in six (14%) of the cases. The incidence of xylazine detection in our study is consistent with the sharp rise reported in other recent studies. In a recent five years, New Jersey saw more than a 3100% increase in the positivity rate of xylazine, detected most often in combination with heroin and fentanyl analytes in substances seized by authorities [51]. In 2019, xylazine was detected in 31% of opioid-related toxicity fatalities in Philadelphia, a city that is directly adjacent to the South New Jersey region [52–54].

Medications commonly used to treat depression and psychiatric conditions were detected in 12 (29%) of the cases, though it is unclear if the antidepressants were obtained from a physician as a measure of clinical care, as medical records were not available to the researchers of this study. Although antidepressants are subject to misuse by those with OUD, they also associate with increased retention to buprenorphine treatment for OUD [55, 56]. However, there was little evidence that the decedents in our study were receiving medication-assisted treatment for OUD.

To test the hypothesis that individuals with OUD who are in clinical care may present with a lesser degree of co-occurring substance use than those who are not in clinical care, we compared the average number of putative illicit substances besides opioids (classifications of dissociatives, psychoactive bath salts, sedatives, and stimulants except for caffeine, nicotine, and cotinine) between decedents with and without evidence of antidepressant/antianxiety medications in their toxicology results. However, there was no significant difference in the average number of non-opioid illicit substances detected between the two groups (Mann-Whitney-Wilcoxon p value of 0.1).

Our results show some evidence that opioid-toxicity reversal medication was administered to decedents, although the timing of the medication dose with respect to the toxicity reaction is unclear. Naloxone, often used in attempt to reverse an opioid toxicity reaction at the time of first response, was detected in eight (19%) of the cases. The overall efficacy of naloxone against fentanyl and fentanyl analogs is questionable given the rapid onset time of toxicity for HPSO and the very brief time window for reversal of toxicity events [57]. Although naloxone is effective as an opioid toxicity antidote, it is unclear the degree to which its efficacy may be reduced when other substances (e.g., stimulants and sedatives) are also present [54, 57]. In addition, due to the rise in the use of high potency opioids such as fentanyl, the Centers for Disease Control and Prevention recently recommended that healthcare providers counsel patients that an increased number and/or higher doses of naloxone may be needed to counteract a toxicity event [8]. The half-life of naloxone is approximately 1.0–1.5 hours, and the half-life of some high-potency opioids far exceeds that, making rebound toxicity of the opioids a possibility once naloxone concentrations drop [58–60]. Naloxone is also a component of suboxone, a combined medication that includes buprenorphine; however in this preparation, naloxone may not be absorbed into the systemic circulation in quantities sufficient to be detected on a drug screen [61, 62]. Also of note is that use of naloxone to reverse a toxicity event may lead to a recurrence of toxicity symptoms later as well as new psychiatric, medical, and toxicological conditions [8, 63, 64].

Buprenorphine and methadone, currently approved MOUD treatment drugs, were detected in only three of the decedent toxicology samples. The small number of cases with evidence of MOUD (7%) lends support to concerns about limited access to medication treatment and clinical care for those living with OUD, and about underuse and low retention rates of MOUD particularly among those who engage in co-occurring substance use [25, 65, 66]. The toxicology report from only one (2%) of the 42 decedents showed the presence of free norbuprenorphine, the metabolite of buprenorphine, a primary MOUD [67–70]. Detection of methadone, a full opioid agonist, was similarly limited, present in only two (5%) of the cases, and in one among them at a potentially fatal concentration to indicate possible misuse outside of clinical care [67, 71, 72].

Potential explanations for the low incidence of MOUD in this study are limited access to MOUD, low adherence rates for MOUD, and the stigma of seeking treatment [73–77]. While New Jersey has in recent years been expanding access to MOUD within its jails, it is less clear to what extent access has expanded outside of the criminal justice system [78]. Moreover, co-occurring SUD is associated with lower MOUD initiation and retention rates, and aligns with our study’s evidence showing a low rate of MOUD and a high rate of co-occurring substance use [66, 67, 79–81]. The one case in our study showing evidence of buprenorphine had fentanyl and its metabolites, norfentanyl and 4-ANPP present, but no evidence of co-occurring substance use except for nicotine and caffeine.

The low incidence of detected MOUD, buprenorphine or methadone, in the current study may be explained because these drugs protect against death from opioid toxicity when taken at the correct dosage. Indeed, prior studies show that MOUD significantly decrease the occurrence of toxicity events among persons with OUD, with additional reductions in the likelihood of toxicity events associated with longer durations of medication treatment for OUD up to 12 months [82]. Buprenorphine is less likely to be misused and lead to toxicity events compared to methadone [70, 83]. This difference arises because buprenorphine is a partial opioid receptor agonist that is limited by a ceiling effect unlike methadone, a full agonist that induces maximal opioid effects including euphoria but also adverse effects such as respiratory depression (but to a lesser degree than most opioids of abuse) [68, 83, 84]. According to one report, in similar doses, methadone can be more effective than buprenorphine, but a medium dose of buprenorphine (8–15 mg) is as effective as a lower dose of methadone in suppressing opioid use [83]. While both MOUD are effective at reducing the risk of mortality compared to no treatment, methadone is associated with higher retention rates than buprenorphine; however, during induction and after leaving treatment, mortality rates are higher with methadone than with buprenorphine, and co-occurring substance use is a contributing factor to methadone-related death [85–88]. Buprenorphine prescribing is increasing in New Jersey, the first state to introduce it at the time of emergency response, immediately following reversal of an opioid toxicity event. This practice provides a “soft-landing” from opioid withdrawal symptoms, initiates MOUD treatment, and increases retention rates in recovery [89–92].

## Conclusions

Expanded toxicology reports of 42 decedents who expired from opioid-related toxicity in the South New Jersey region from March 2019 through April 2021 were analyzed as part of the Camden Opioid Research Initiative. The HPSO, fentanyl, was found in 41 (98%) of the toxicology cases, and either buprenorphine or methadone was found in only three (7%) of the cases, affirming a high incidence rate of opioid-toxicity events involving HPSO and a low incidence rate of MOUD among individuals who die from suspected HPSO toxicity. Naloxone, a toxicity reversal medication to be administered at the time of first response, was detected in eight (19%) of the cases. The outcome suggests that the naloxone dose may have been insufficient to counteract the HPSO or the HPSO when combined with an array of other detected compounds, as evidence of co-occurring stimulant use was detected in all decedent samples, and sedative use in nearly half of decedent samples, often coinciding with non-HPSO opioids and non-opioid analgesics. Alternatively, failure to detect naloxone in some samples may be due to its short half-life.

To confront the increased risk of opioid toxicity death presented by HPSO and co-occurring substance use, a multi-faceted strategy is needed. Guided by the data observed in this study, an effective response to reduce the likelihood of a fatal toxicity event would include special consideration for co-occurring substance use in both treatment and emergency response with respect to naloxone dosing, increased and immediate availability of naloxone to the general population, elimination of MOUD stigma, and increased access to MOUD and clinical care.

## Supporting information

S1 Fig Detected compounds grouped by class per case. Frequency of toxicology compounds by class, in descending order, detected per case in 42 postmortem samples of femoral blood collected from opioid-related toxicity cases submitted to the CORI Biobank. Each bar on the x-axis is a detected compound class, the y-axis is the case incidence count, and the shading indicates the number of compounds detected for a given class per case.
(TIF)

S1 Table Table of detected compounds. Compounds (N = 77) detected in expanded toxicology panels among postmortem blood samples collected from 42 opioid-related drug toxicity death cases as part of the Camden Opioid Research Initiative. Detected concentrations are shown numerically where available, else, reported as Positive.
(DOCX)

S1 File Listing of tested compounds. A full listing of the more than 360 analytes tested by the two panels, 8054B and 8092B, at NMS Labs.
(XLSX)

S2 File Toxicology results data set. Double-blinded raw data of toxicology testing results used for analysis in this study.
(XLSX)
