# Supplementary figures and images for: Postmortem toxicology findings from the Camden Opioid Research Initiative
Source: PLoS One. 2023 Nov 1;18(11):e0292674. doi: 10.1371/journal.pone.0292674 (PMC10619848; doi:10.1371/journal.pone.0292674)

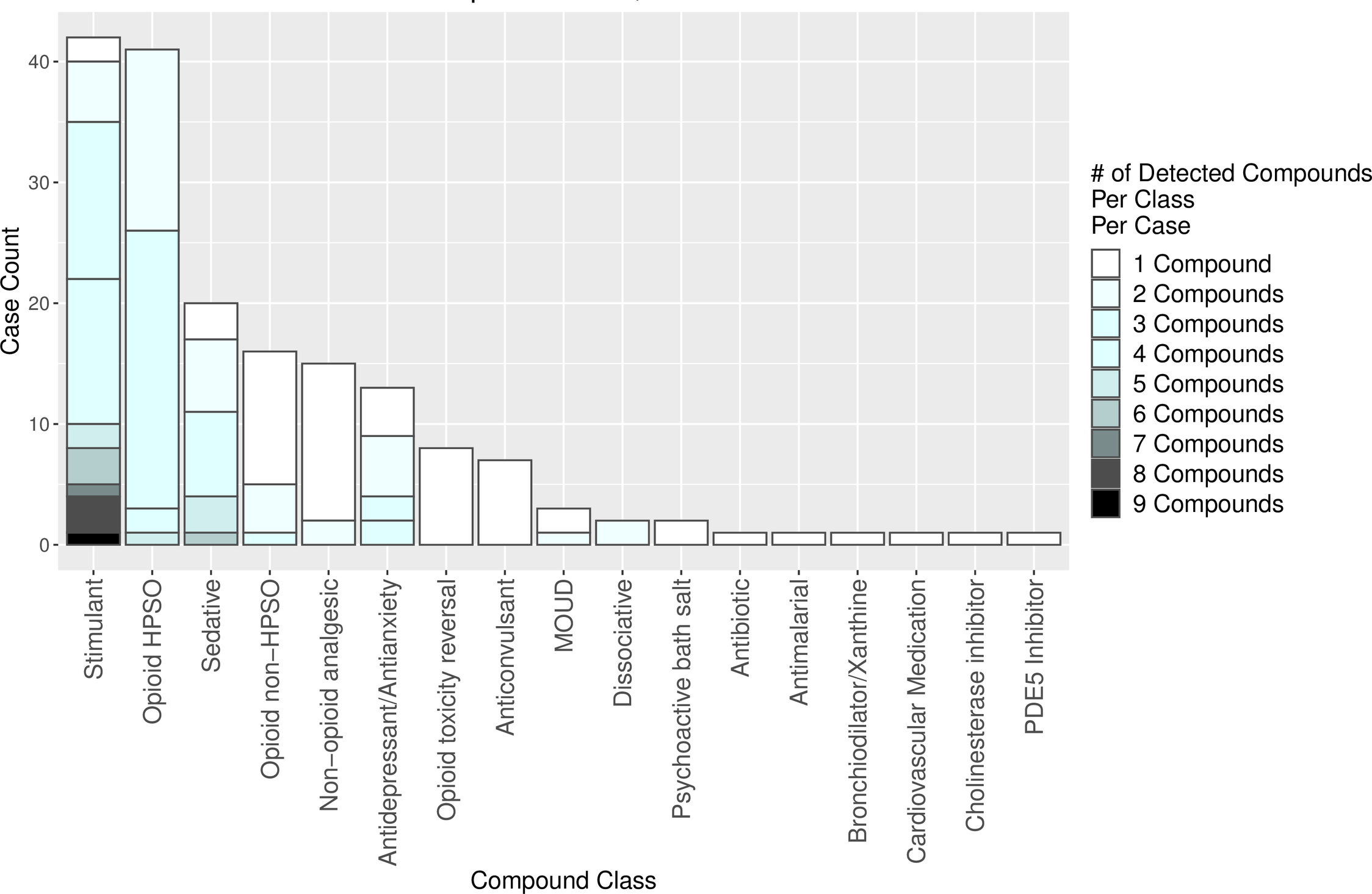

Supplement: S1 Fig — Frequency of toxicology compounds by class, in descending order, detected per case in 42 postmortem samples of femoral blood collected from opioid-related toxicity cases submitted to the CORI Biobank. Each bar on the x-axis is a detected compound class, the y-axis is the case incidence count, and the shading indicates the number of compounds detected for a given class per case. (TIF) [file pone.0292674.s001.tif]
