# Supplementary material for: Postmortem toxicology findings from the Camden Opioid Research Initiative
Source: PLoS One. 2023 Nov 1;18(11):e0292674. doi: 10.1371/journal.pone.0292674 (PMC10619848; doi:10.1371/journal.pone.0292674)
Supplement: S1 Table — Compounds (N = 77) detected in expanded toxicology panels among postmortem blood samples collected from 42 opioid-related drug toxicity death cases as part of the Camden Opioid Research Initiative. Detected concentrations are shown numerically where available, else, reported as Positive. (DOCX) [file pone.0292674.s002.docx]

| **Compound Name** | **Metabolite Relationships** | **Classification for Study** | **Classification Studied for Co-occurring SUD** | **Reporting Limit(s)** | **# Cases Detected (%)** | **Detected Amount Median, [min, max] Unit** |
| --- | --- | --- | --- | --- | --- | --- |
| 11-Hydroxy Delta-9 THC | 11-Hydroxy Delta-9 THC is an active intermediate metabolite of tetrahydrocannabinol (THC) | Sedative | Y | 1.00 ng/mL | 3 (7%) | 1.8, [1.6, 3.9] ng/mL |
| 2-Furanylfentanyl |  | Opioid HPSO | Y | 0.05 ng/mL | 1 (2%) | 0.1, [0.1, 0.1] ng/mL |
| 4-ANPP | 4-ANPP is a fentanyl metabolite. | Opioid HPSO | Y | 0.1 – 1.0 ng/mL | 26 (62%) | 1.6, [0.3, 11.0] ng/mL |
| 6-Monoacetylmorphine - Free | 6-monoacetylmorphine (6-MAM) is the 6-monoacetylated form of morphine. | Opioid non-HPSO | Y | 1.0 ng/mL | 4 (10%) | 1.3, [1.0, 2.4] ng/mL |
| 7-Amino Clonazepam | 7-Amino-Clonazepam is a metabolite of clonazepam, a benzodiazepine drug. | Sedative | Y | 5.0 ng/mL | 4 (10%) | 32.0, [6.2, 290.0] ng/mL |
| Acetaminophen |  | Non-opioid analgesic |  | 0.5 – 20.0 ng/mL | 5 (12%) | 1.4, [0.6, 68.0] mcg/mL |
| Acetyl Fentanyl |  | Opioid HPSO | Y | 0.1 – 0.2 ng/mL | 4 (10%) | 4.9, [0.2, 28.0] ng/mL |
| Alprazolam |  | Antidepressant/Antianxiety |  | 5.0 ng/mL | 5 (12%) | 25.0, [17.0, 100.0] ng/mL |
| Amlodipine |  | Cardiovascular Medication |  | 10.0 ng/mL | 1 (2%) | 50.0, [50.0, 50.0] ng/mL |
| Amphetamine |  | Stimulant | Y | 5.0 ng/mL | 5 (12%) | 52.0, [9.7, 140.0] ng/mL |
| Aripiprazole |  | Antidepressant/Antianxiety |  | 20.0 ng/mL | 1 (2%) | 28.0, [28.0, 28.0] ng/mL |
| Benzoylecgonine | Benzoylecgonine is an inactive metabolite of cocaine. | Stimulant | Y | 50.0 – 100.0 ng/mL | 13 (31%) | 1000.0, [54.0, 2200.0] ng/mL |
| Beta-Phenethylamine |  | Psychoactive bath salt | Y | 250.0 – 500.0 ng/mL | 2 (5%) | Positive |
| Bupropion |  | Antidepressant/Antianxiety |  | 10.0 ng/mL | 1 (2%) | 35.0, [35.0, 35.0] ng/mL |
| Buspirone |  | Antidepressant/Antianxiety |  | 0.5 ng/mL | 1 (2%) | 40.0, [40.0, 40.0] ng/mL |
| Caffeine |  | Stimulant |  | 0.1 – 0.4 ng/mL | 37 (88%) | Positive |
| Citalopram / Escitalopram |  | Antidepressant/Antianxiety |  | 5.0 ng/mL | 1 (2%) | 48.0, [48.0, 48.0] ng/mL |
| Clonazepam | A benzodiazepine drug. | Sedative | Y | 2.0 ng/mL | 1 (2%) | 3.0, [3.0, 3.0] ng/mL |
| Cocaethylene | Byproduct of concurrent consumption of alcohol and cocaine. | Sedative | Y | 20.0 ng/mL | 3 (7%) | 52.0, [26.0, 78.0] ng/mL |
| Cocaine | Cocaine is metabolized to the inactive compounds benzoylecgonine, ecgonine methyl ester, and ecgonine. | Stimulant | Y | 20.0 ng/mL | 5 (12%) | 55.0, [48.0, 160.0] ng/mL |
| Codeine - Free |  | Opioid non-HPSO | Y | 5.0 ng/mL | 2 (5%) | 8.0, [7.3, 8.6] ng/mL |
| Cotinine | Cotinine is a metabolite of nicotine | Stimulant |  | 200.0 – 400.0 ng/mL | 33 (79%) | Positive |
| Cyclobenzaprine |  | Sedative | Y | 1.0 ng/mL | 2 (5%) | 45.0, [1.9, 88.0] ng/mL |
| Delta-9 Carboxy THC | Delta-9-carboxy-THC (THCC) is the inactive metabolite of THC. | Sedative | Y | 5.0 ng/mL | 6 (14%) | 8.6, [6.6, 28.0] ng/mL |
| Delta-9 THC | Delta-9-THC is an active metabolite of THC | Sedative | Y | 0.5 ng/mL | 7 (17%) | 3.7, [1.6, 15.0] ng/mL |
| Desmethylsertraline | Desmethylsertraline is the principal metabolite of sertraline | Antidepressant/Antianxiety |  | 20.0 ng/mL | 2 (5%) | 94.5, [29.0, 160.0] ng/mL |
| Dextro / Levo Methorphan |  | Dissociative | Y | 5.0 ng/mL | 2 (5%) | 345.0, [290.0, 400.0] ng/mL |
| Dextrorphan / Levorphanol |  | Dissociative | Y | 2.0 ng/mL | 2 (5%) | 87.5, [35.0, 140.0] ng/mL |
| Diazepam |  | Sedative | Y | 20.0 ng/mL | 2 (5%) | 98.0, [26.0, 170.0] ng/mL |
| Dicyclomine |  | Sedative | Y | 1.0 ng/mL | 1 (2%) | 5.2, [5.2, 5.2] ng/mL |
| Diphenhydramine |  | Sedative | Y | 50.0 ng/mL | 2 (5%) | 115.0, [50.0, 180.0] ng/mL |
| Donepezil |  | Cholinesterase inhibitor |  | 5.0 ng/mL | 1 (2%) | 120.0, [120.0, 120.0] ng/mL |
| Doxylamine |  | Sedative | Y | 100.0 ng/mL | 1 (2%) | 400.0, [400.0, 400.0] ng/mL |
| EDDP | EDDP (2-ethylidene-1,5-dimethyl-3,3-diphenylpyrrolidine) is the primary inactive metabolite of methadone. | MOUD |  | 20.0 ng/mL | 1 (2%) | 180.0, [180.0, 180.0] ng/mL |
| Ephedrine |  | Stimulant | Y | 5.0 ng/mL | 1 (2%) | 9.1, [9.1, 9.1] ng/mL |
| Ethanol | Alcohol. | Sedative | Y | 10.0 ng/mL | 7 (17%) | 61.0, [13.0, 401.0] mg/dL |
| Ethylecgonine | Cocaethylene metabolite, formed when cocaine and ethyl alcohol are used simultaneously. | Sedative, Stimulant | Y | 100.0 ng/mL | 4 (10%) | Positive |
| Fentanyl |  | Opioid HPSO | Y | 0.1 – 1.0 ng/mL | 41 (98%) | 16.0, [1.3, 73.0] ng/mL |
| Fluoxetine |  | Antidepressant/Antianxiety |  | 20.0 ng/mL | 2 (5%) | 350.0, [220.0, 480.0] ng/mL |
| Gabapentin |  | Anticonvulsant |  | 1.0 mcg/mL | 5 (12%) | 10.0, [7.4, 23.0] mcg/mL |
| Guaifenesin |  | Sedative | Y | 0.2 mcg/mL | 1 (2%) | 3.1, [3.1, 3.1] mcg/mL |
| Hydroxybupropion |  | Antidepressant/Antianxiety |  | 100.0 ng/mL | 1 (2%) | 1100.0, [1100.0, 1100.0] ng/mL |
| Ibuprofen |  | Non-opioid analgesic |  | 3.0 – 20.0 mcg/mL | 8 (19%) | Positive |
| Lamotrigine |  | Anticonvulsant |  | 0.2 mcg/mL | 2 (5%) | 2.9, [0.5, 5.2] mcg/mL |
| Levamisole | Levamisole is an imidazothiazole derivative | Stimulant | Y | 5.0 – 250.0 ng/mL | 2 (5%) | Positive |
| mCPP | Meta-chlorophenylpiperazine (mCPP) is an active metabolite of the prescription antidepressants Trazodone, and Nefazodone. | Antidepressant/Antianxiety |  | 0.05 mcg/mL | 1 (2%) | 0.1, [0.1, 0.1] mcg/mL |
| Methadone | Major metabolites of methadone include EDDP and EMDP. | MOUD |  | 20.0 ng/mL | 2 (5%) | 750.0, [100.0, 1400.0] ng/mL |
| Methamphetamine | Amphetamine and norephedrine (phenylpropanolamine) are metabolites of methamphetamine. d- | Stimulant | Y | 5.0 ng/mL | 5 (12%) | 320.0, [12.0, 1900.0] ng/mL |
| Methylecgonine | Methylecgonine is a metabolite of cocaine. | Stimulant | Y | 100.0 ng/mL | 3 (7%) | Positive |
| Metoprolol |  | Stimulant | Y | ng/mL | 2 (5%) | 111.5, [53.0, 170.0] ng/mL |
| Mitragynine |  | Non-opioid analgesic |  | ng/mL | 1 (2%) | 72.0, [72.0, 72.0] ng/mL |
| Morphine - Free |  | Opioid non-HPSO | Y | ng/mL | 14 (33%) | 47.0, [6.3, 140.0] ng/mL |
| Naloxone |  | Opioid toxicity reversal |  | 1.0 – 2.0 ng/mL | 8 (19%) | Positive |
| Naproxen |  | Non-opioid analgesic |  | 3.0 mcg/mL | 3 (7%) | 20.0, [3.8, 20.0] mcg/mL |
| Nicotine |  | Stimulant |  | 5.0 – 25.0 ng/mL | 33 (79%) | 210.0, [200.0, 270.0] ng/mL, and Positive |
| Norbuprenorphine - Free | Norbuprenorphine is the metabolite of buprenorphine | MOUD |  | 1.0 ng/mL | 1 (2%) | 2.4, [2.4, 2.4] ng/mL |
| Norcyclobenzaprine | Norcyclobenzaprine is one of the major metabolites of cyclobenzaprine | Sedative | Y | 10.0 ng/mL | 2 (5%) | Positive |
| Nordiazepam | Nordiazepam is an active metabolite of several benzodiazepines, including diazepam (Valium®) and chlordiazepoxide (Librium®). | Sedative | Y | 20.0 ng/mL | 3 (7%) | 130.0, [34.0, 1000.0] ng/mL |
| Norfentanyl | Norfentanyl is the primary inactive metabolite of the synthetic narcotic analgesic fentanyl. | Opioid HPSO | Y | 0.2 – 0.4 ng/mL | 39 (93%) | 2.2, [0.2, 20.0] ng/mL |
| Norfluoxetine | Norfluoxetine is an active metabolite of fluoxetine | Antidepressant/Antianxiety |  | 20.0 – 40.0 ng/mL | 3 (7%) | 380.0, [200.0, 490.0] ng/mL |
| O-Desmethylvenlafaxine | O-Desmethylvenlafaxine is a venlafaxine metabolite. | Antidepressant/Antianxiety |  | 20.0 ng/mL | 1 (2%) | 930.0, [930.0, 930.0] ng/mL |
| Olanzapine |  | Antidepressant/Antianxiety |  | 3.0 ng/mL | 2 (5%) | 280.0, [170.0, 390.0] ng/mL |
| Oxycodone - Free |  | Opioid non-HPSO | Y | 10.0 ng/mL | 2 (5%) | 25.5, [20.0, 31.0] ng/mL |
| para-Fluoroisobutyrylfentanyl |  | Opioid HPSO | Y | 0.05 ng/mL | 1 (2%) | 0.3, [0.3, 0.3] ng/mL |
| Paroxetine |  | Antidepressant/Antianxiety |  | 10.0 ng/mL | 1 (2%) | 27.0, [27.0, 27.0] ng/mL |
| Phenylpropanolamine |  | Stimulant | Y | 20.0 ng/mL | 1 (2%) | 54.0, [54.0, 54.0] ng/mL |
| Quetiapine |  | Antidepressant/Antianxiety |  | 50.0 ng/mL | 1 (2%) | 54.0, [54.0, 54.0] ng/mL |
| Quinine |  | Antimalarial |  | 0.05 mcg/mL | 1 (2%) | Positive |
| Sertraline |  | Antidepressant/Antianxiety |  | 10.0 ng/mL | 2 (5%) | 22.5, [11.0, 34.0] ng/mL |
| Tadalafil |  | PDE5 Inhibitor |  | 10.0 ng/mL | 1 (2%) | 37.0, [37.0, 37.0] ng/mL |
| Theobromine |  | Stimulant |  | 5.0 – 10.0 mcg/mL | 19 (45%) | Positive |
| Theophylline |  | Bronchodilator |  | 0.5 mcg/mL | 1 (2%) | Positive |
| Topiramate |  | Sedative | Y | 200.0 ng/mL | 2 (5%) | 3150.0, [1700.0, 4600.0] ng/mL |
| Trazodone |  | Antidepressant/Antianxiety |  | 0.05 mcg/mL | 2 (5%) | 0.3, [0.2, 0.4] mcg/mL |
| Trimethoprim |  | Antibiotic |  | 1.0 mcg/mL | 1 (2%) | Positive |
| Venlafaxine |  | Antidepressant/Antianxiety |  | 20.0 ng/mL | 1 (2%) | 120.0, [120.0, 120.0] ng/mL |
| Xylazine |  | Sedative | Y | 5.0 ng/mL | 6 (14%) | 44.5, [8.3, 100.0] ng/mL |
